# Pericytes as a Source of Osteogenic Cells in Bone Fracture Healing

**DOI:** 10.3390/ijms20051079

**Published:** 2019-03-02

**Authors:** Sopak Supakul, Kenta Yao, Hiroki Ochi, Tomohito Shimada, Kyoko Hashimoto, Satoko Sunamura, Yo Mabuchi, Miwa Tanaka, Chihiro Akazawa, Takuro Nakamura, Atsushi Okawa, Shu Takeda, Shingo Sato

**Affiliations:** 1 Department of Physiology and Cell Biology, Tokyo Medical and Dental University, Graduate School, Tokyo 113-8519, Japan; 140521ms@tmd.ac.jp (S.S.); 130921ms@tmd.ac.jp (K.Y.); ochi.phy2@tmd.ac.jp (H.O.); 150531ms@tmd.ac.jp (T.S.); 110682ms@tmd.ac.jp (K.H.); sunphy2@tmd.ac.jp (S.S.); 2 Department of Biochemistry and Biophysics, Tokyo Medical and Dental University, Graduate School, Tokyo 113-8510, Japan; yomabuchi.bb@tmd.ac.jp (Y.M.); c.akazawa.bb@tmd.ac.jp or chihiroakazawa@gmail.com (C.A.); 3 Division of Carcinogenesis, The Cancer Institute, Japanese Foundation for Cancer Research (JFCR), Tokyo 135-8550, Japan; miwa.tanaka@jfcr.or.jp (M.T.); nakamurat@jfcr.or.jp (T.N.); 4 Department of Orthopaedic Surgery, Tokyo Medical and Dental University, Graduate School, Tokyo 113-8519, Japan; okawa.orth@tmd.ac.jp; 5 Division of Endocrinology, Toranomon Hospital Endocrine Center, Tokyo 105-8470, Japan; takeda.phy2@tmd.ac.jp

**Keywords:** pericyte, osteogenic differentiation, bone fracture healing

## Abstract

Pericytes are mesenchymal cells that surround the endothelial cells of small vessels in various organs. These cells express several markers, such as NG2, CD146, and PDGFRβ, and play an important role in the stabilization and maturation of blood vessels. It was also recently revealed that like mesenchymal stem cells (MSCs), pericytes possess multilineage differentiation capacity, especially myogenic, adipogenic, and fibrogenic differentiation capacities. Although some previous studies have reported that pericytes also have osteogenic potential, the osteogenesis of pericytes can still be further elucidated. In the present study, we established novel methods for isolating and culturing primary murine pericytes. An immortalized pericyte line was also established. Multilineage induction of the pericyte line induced osteogenesis, adipogenesis, and chondrogenesis of the cells in vitro. In addition, pericytes that were injected into the fracture site of a bone fracture mouse model contributed to callus formation. Furthermore, in vivo pericyte-lineage-tracing studies demonstrated that endogenous pericytes also differentiate into osteoblasts and osteocytes and contribute to bone fracture healing as a cellular source of osteogenic cells. Pericytes can be a promising therapeutic candidate for treating bone fractures with a delayed union or nonunion as well as bone diseases causing bone defects.

## 1. Introduction

Bone fracture is one of the most frequent problems found in orthopedic wards around the world (406 per 100,000 people per year [1]). In addition, the healing process can take up to 6–12 weeks. Moreover, despite the long duration of healing, previous studies reported that fracture healing failed in 10–20% of all fractures, resulting in nonunions and potentially causing severe disabilities [2].

Bone fracture healing is complicated because the process comprises many specific biological pathways involving the acute inflammatory response, recruitment of mesenchymal stem cells (MSCs), primary cartilaginous callus formation, revascularization, and calcification [3]. Stem cells, especially MSCs, have been reported to play an important role in these processes. MSCs are well known for their multilineage potential, including osteogenic and chondrogenic differentiation capabilities [4]. In response to bone fracture, MSCs are recruited to the fracture site and contribute to bone fracture healing [5]. However, there is an argument regarding the homogeneity of MSCs since MSCs usually contain a heterogeneous population of cells. In addition, MSCs are not the only source of osteogenic cells. Several previous studies reported other possible cell sources that could be converted to osteogenic cells, for example, endothelial cells [6], endoneurial progenitor cells [7], and hematopoietic stem cells [8].

Pericytes are defined as the cells that surround the endothelial cells of capillaries and microvessels [9]. Pericytes express several markers, such as neuron glial antigen 2 (NG2), CD146, and PDGFRβ. Functionally, pericytes have been shown to contribute to the stabilization and maturation of blood vessels [10,11]. A recent study also suggested pericytes as a cellular source of certain mesenchymal tissue neoplasms [12]. It has also been recently revealed that like MSCs, pericytes possess multilineage differentiation capacities, especially myogenic, adipogenic, or fibrogenic differentiation [13,14,15]. Although some previous studies have reported that pericytes also have osteogenic differentiation potential, the osteogenesis of pericytes as well as their role in the bone fracture healing process can still be further elucidated.

In the present study, we established novel methods for the isolation and culture of primary murine pericytes. We also generated an immortalized pericyte line and showed the osteogenic differentiation potential of the cells in vitro and in vivo. Furthermore, in vivo pericyte-lineage-tracing studies have demonstrated that endogenous pericytes also differentiate into osteoblasts and osteocytes and contribute to bone fracture healing as a cellular source of osteogenic cells.

## 2. Results

### 2.1. A Novel Method for Isolating and Culturing Primary Pericytes

**Figure 1:**
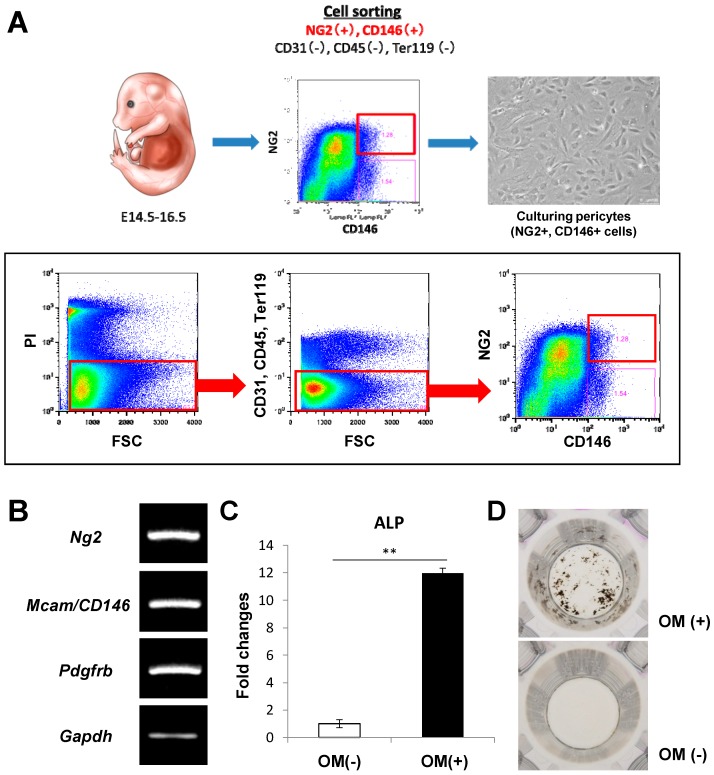
Isolation of primary pericytes from mouse embryos and their osteogenic differentiation capacity. (**A**) Primary pericytes were isolated from mouse embryos at 14.5–16.5 dpc using flow cytometry. NG2+, CD146+, CD31−, CD45−, and Ter119− cells were sorted and cultured. (**B**) PCR analysis showing the expression of the pericyte markers *Ng2*, *Mcam/Cd146*, and *Pdgfrb* in the cultured cells. An alkaline phosphatase (ALP) activity assay (**C**) and von Kossa staining (**D**) showing that osteogenic differentiation of the sorted pericytes was induced after 6 days of osteogenic induction. OM: osteogenic induction medium. All the data are means ± SDs (*n* = 3). ** *p* < 0.01 by Student’s *t*-test (**C**).

**Figure 2:**
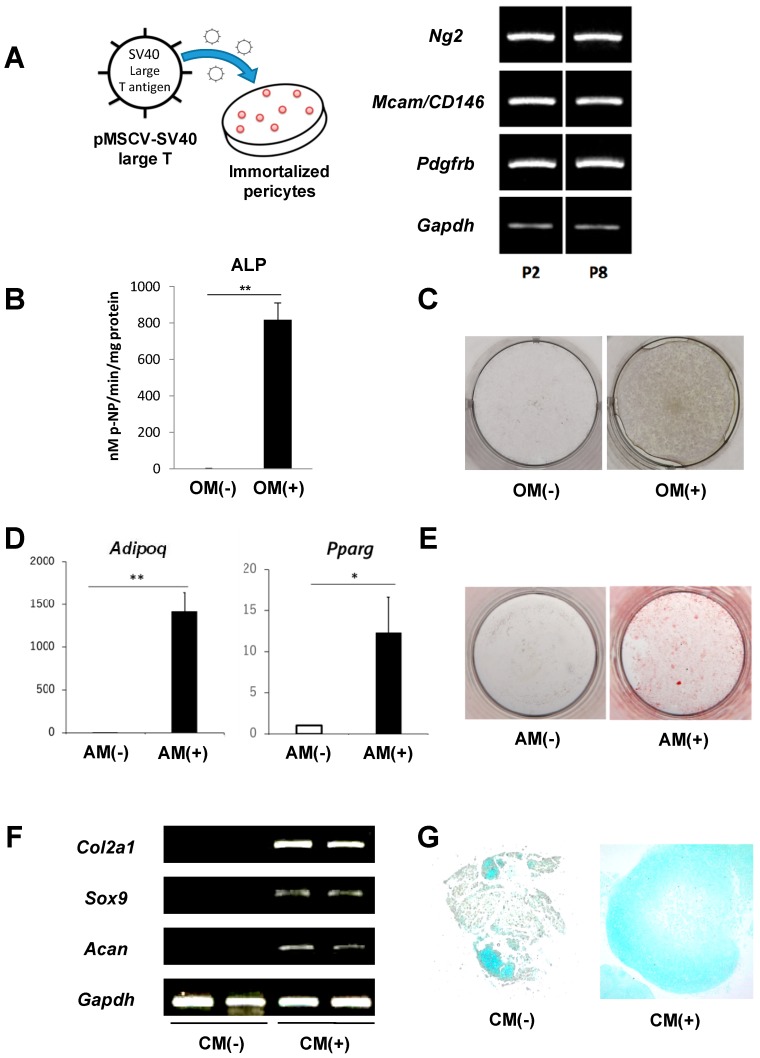
Immortalization of primary pericytes and their multilineage differentiation ability. (**A**) SV40 large T antigen was transduced into the sorted primary pericytes to immortalize the cells. PCR analysis showing the expression levels of the pericyte markers *Ng2*, *Mcam/CD146*, and *Pdgfrb* after the immortalized cells were passaged two times (P2) and eight times (P8). An ALP activity assay (**B**) and von Kossa staining (**C**) showing that osteogenic induction remarkably increased the ALP activity of cells and induced mineralized nodules. (**D**) Quantitative PCR analyses showing the significantly increased expression of the adipogenic markers *Adipoq* and *Pparg* in adipogenic-induced pericytes. (**E**) Oil Red O staining showing that adipogenic induction promoted lipid droplet formation of the cells. (**F**) The expression of chondrocyte markers *Col2a1*, *Sox9*, and *Acan* was upregulated in the chondrogenic-induced pericytes that were cultured by a pellet culture system. (**G**) Representative alcian blue staining of the pellets with chondrogenic induction showing an abundance of extracellular cartilage matrix. OM: osteogenic induction medium. AM: adipogenic induction medium. CM: chondrogenic induction medium. All the data are means ± SDs (*n* = 3). * *p* < 0.05, ** *p* < 0.01 by Student’s *t*-test (**B**,**D**).

Pericytes express several markers, such as NG2, CD146, and PDGFRβ. To isolate a more homogenous population of pericytes, NG2 and CD146 double-positive cells were sorted from mouse embryos at 14.5–16.5 dpc by fluorescence-activated cell sorting (FACS) analysis. Cells positive for CD31 (a marker of endothelial cells), CD45 (a marker of hematopoietic cells), or Ter119 (a marker of red blood cells) were eliminated (Figure 1A). Sorted cells were seeded on a 48-well plate and successfully expanded in the indicated culture media. PCR analysis confirmed that the sorted and cultured pericytes express the pericyte markers *Ng2*, *Mcam/CD146*, and *Pdgfrb* (Figure 1B). In addition, to investigate the osteogenic differentiation potential of the cells, the sorted cells were cultured in osteogenic induction medium. A 6-day osteogenic induction period significantly promoted the osteogenic differentiation of the pericytes, as shown by the increase in alkaline phosphatase (ALP) activity (Figure 2C). After a 9-day induction, von Kossa staining was performed to investigate the matrix mineralization ability of the cells. The osteogenic induction extensively induced mineralized nodule formation of the sorted pericytes (Figure 2D).

### 2.2. Immortalization of Primary Pericytes and Multilineage Differentiation Capacity In Vitro

The growth of the sorted primary pericytes became slow with repeated passages, and the cells reached senescence at passage five or six. To immortalize the isolated pericytes, the cells were transduced with SV40 large T antigen by a retroviral transduction system (Figure 2A). The transduction did not affect cell morphology. In addition, the transduced cells also expressed pericyte markers, *Ng2*, *Mcam/CD146*, and *Pdgfrb*, even after the passage was repeated more than eight times (Figure 2A), suggesting that the immortalized pericytes maintained their characteristics. Next, to investigate the multilineage potential of the immortalized pericytes, the cells were cultured in osteogenic, adipogenic, and chondrogenic induction medium. An ALP activity assay and von Kossa staining showed that osteogenic induction remarkably increased ALP activity and promoted mineralized nodule formation in the cells (Figure 2B,C). Adipogenesis-induced pericytes showed significantly increased expression levels of *Adipoq* and *Pparg*, which are markers of adipogenesis (Figure 2D). Oil Red O staining also showed that the number of lipid droplets was dramatically increased by adipogenic induction (Figure 2E). Chondrogenic differentiation of the pericytes was studied using a pellet culture system. PCR analysis showed that a four-week chondrogenic induction period increased the expression of chondrogenic markers, such as *Col2a1*, *Sox9*, and *Acan*, in the developed pellets (Figure 2F). Alcian blue staining also showed an abundance of extracellular cartilage matrix in the chondrogenic-induced pellets (Figure 2G). These findings suggest that our immortalized pericytes possess multilineage differentiation capacities similar to that of MSCs.

### 2.3. Pericytes Differentiate into Osteogenic Cells In Vivo

**Figure 3:**
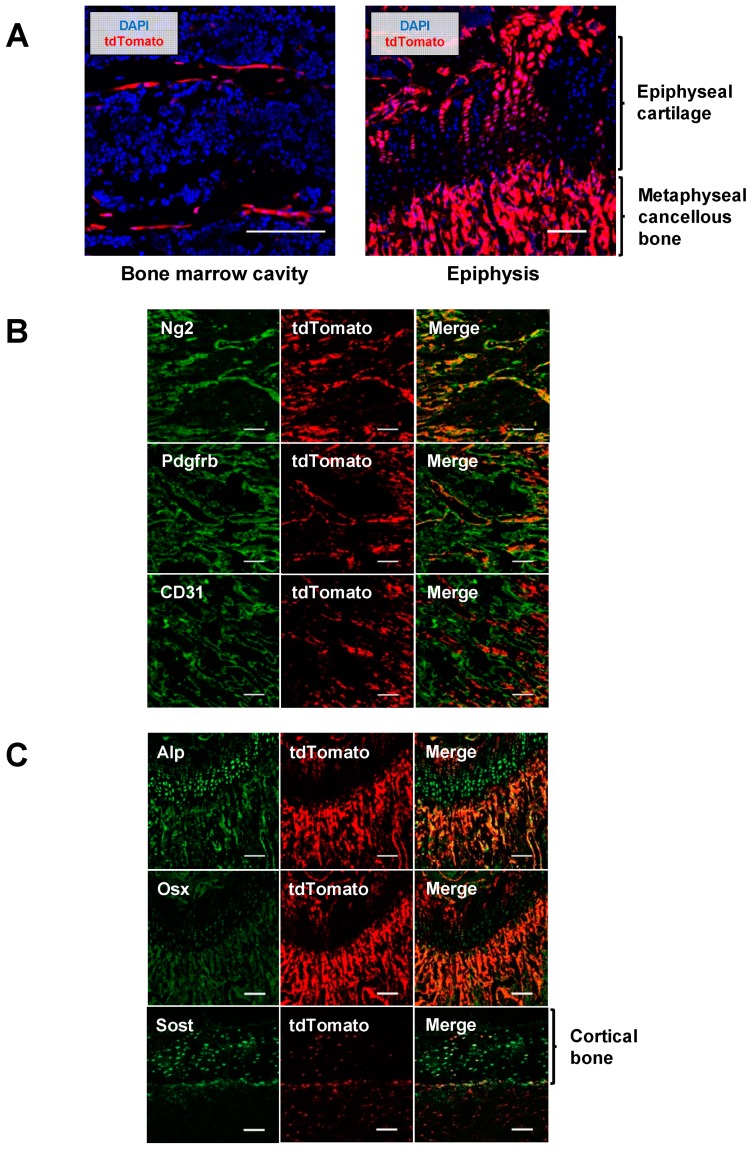
Pericytes differentiated into osteogenic cells in vivo. (**A**) Femurs of 4-week-old *Ng2-Cre;Rosa26R^tdTomato^* mice were harvested, and the distribution of tdTomato-expressing cells was histologically analyzed. Scale bars, 100 μm. (**B**) Immunohistochemical analyses showing that tdTomato-positive cells in the bone marrow cavity coexpressed pericyte markers Ng2 and Pdgfrb but not CD31, an endothelial cell marker. Scale bars, 100 μm. (**C**) tdTomato-expressing cells in the metaphyseal region and in the cortical area coexpressed osteoblast markers, Alp and Osx, and an osteocyte marker, Sost, respectively. Scale bars, 100 μm.

To investigate the fate of pericytes in vivo, *Ng2-Cre;Rosa26R^tdTomato^* mice were generated by crossing a *Ng2-Cre* mouse line with a *Rosa26R^tdTomato^* mouse line. In this cross, Ng2-positive cells and their progenies can be identified as tdTomato-expressing cells. Femurs were harvested from four-week-old *Ng2-Cre;Rosa26R^tdTomato^* mice and histologically analyzed. In the bone marrow cavity of femurs, many tdTomato-expressing cells were lined linearly along blood vessels or trabecular bones (Figure 3A, left). Some chondrocytes in the epiphyseal plate and some bone cells in the metaphyseal region also expressed tdTomato (Figure 3A, right). To characterize these tdTomato-expressing cells, immunohistochemical analyses were performed. tdTomato-expressing perivascular cells coexpressed Ng2 and Pdgfrb, which are markers of pericytes. Additionally, tdTomato-expressing cells did not colocalize with CD31, an endothelial cell marker (Figure 3B), suggesting that pericytes were successfully labeled as tdTomato-expressing cells in this mouse model. Interestingly, tdTomato-positive cells around trabecular bones in the metaphyseal region coexpressed Alp and Osx, which are markers of osteoblasts (Figure 3C), indicating that these osteoblasts originated from Ng2-expressing cells, most likely pericytes. tdTomato-positive cells were also observed in the cortical bone. Immunohistochemical analyses showed that these cells expressed Sost protein (Figure 3C), suggesting that some osteocytes are derived from Ng2-expressing pericytes as well.

### 2.4. Contribution of Implanted Pericytes to Bone Fracture Healing

**Figure 4:**
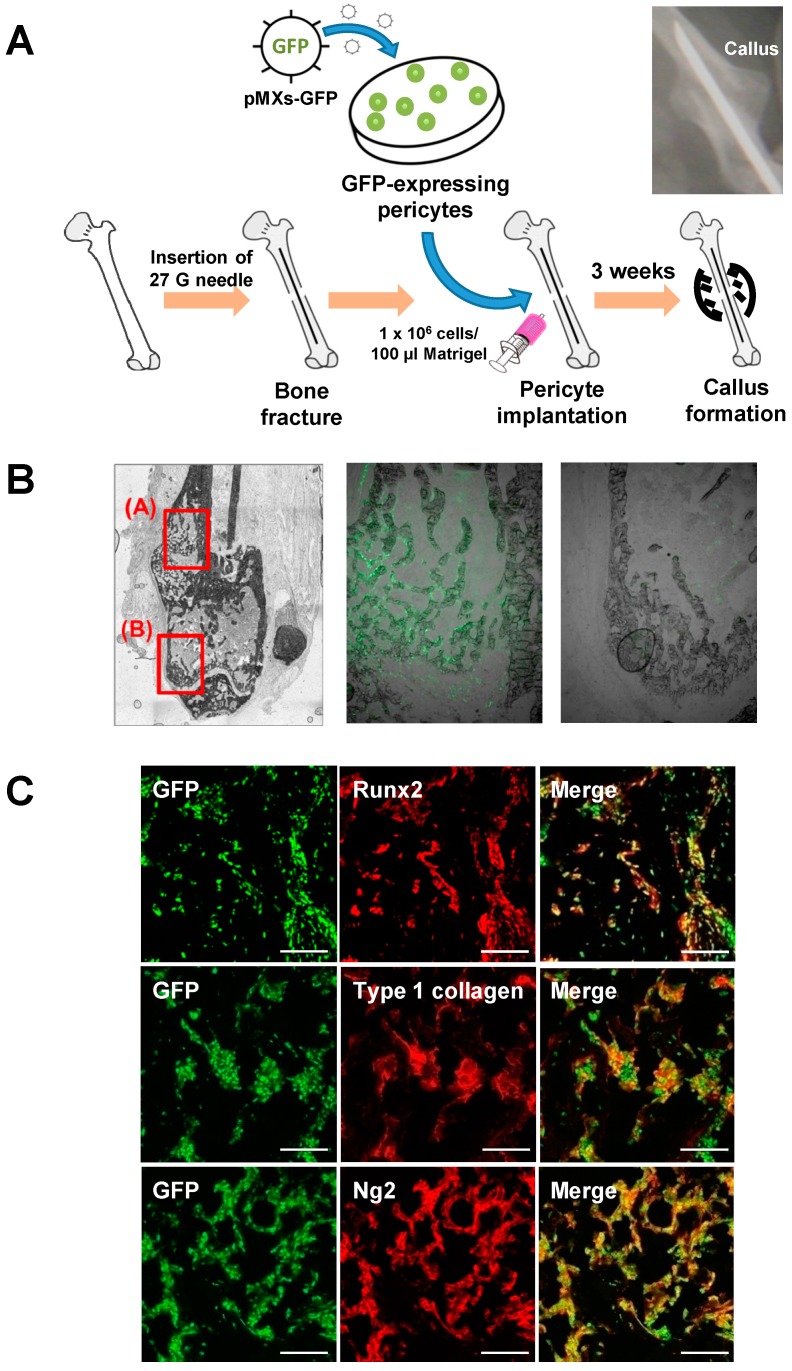
Contribution of implanted pericytes to callus formation during bone fracture healing. (**A**) green fluorescent protein (GFP)-labeled immortalized pericytes (1 × 10^6^ cells/100 µL Matrigel) were injected into the fracture site of the femurs of 12-week-old *BALB/cAJcl-nu/nu* nude mice. (**B**) Histological analysis showing that many GFP-positive cells existed around the newly developed bones. (**C**) Immunohistochemical analyses showing that these GFP-positive cells coexpressed osteoblast markers, Runx2, and type 1 collagen, as well as a pericyte marker, Ng2. Scale bars, 50 μm.

Since pericytes have osteogenic capacity in vitro and in vivo, it is expected that the osteogenic differentiation of pericytes is induced in pathophysiological conditions such as bone fracture. To examine the role of pericytes in bone fracture healing, a femur fracture mouse model was used. To label the immortalized pericytes or their derived cells as green fluorescent protein (GFP)-positive cells, a GFP vector was transduced into the immortalized pericytes using a retroviral system (Figure 4A). The femurs of 12-week-old *BALB/cAJcl-nu/nu* mice were fractured, and GFP-expressing pericytes were injected into the fracture site (Figure 4A). Three weeks after implantation, many GFP-positive cells were located inside the newly developed callus (Figure 4B). In contrast, GFP-positive cells were not observed in any other areas. In addition, immunohistochemical analyses showed that the GFP-expressing cells expressed Runx2 and Col1a1, which are markers for osteoblasts, as well as Ng2 (Figure 4C), suggesting that the implanted pericytes differentiated into osteogenic cells and contributed to the bone fracture healing.

### 2.5. Endogenous Pericytes also Contribute to Bone Fracture Healing In Vivo

**Figure 5:**
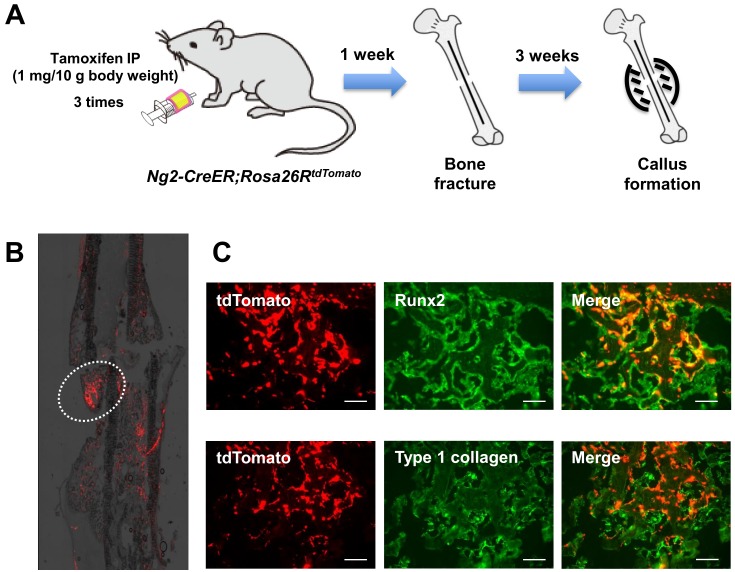
Endogenous pericytes contributed to bone fracture healing in vivo. (**A**) Twenty-week-old *Ng2-CreER;Rosa26R^tdTomato^* mice were injected with tamoxifen 3 times. Then, the femurs were fractured, and the developed callus was analyzed after 3 weeks. (**B**) Histological analysis showing that tdTomato-expressing pericyte-derived cells were recruited inside the newly developed callus. (**C**) Immunohistochemical analyses showing that these tdTomato-positive cells coexpressed osteoblast markers, Runx2, and type 1 collagen. Scale bars, 100 μm.

Using an inducible *Ng2-CreER* mouse line, we generated *Ng2-CreER;Rosa26R^tdTomato^* mice in which tdTomato expression is induced in Ng2-expressing pericytes and their progenies by administration of tamoxifen. To investigate the role of endogenous pericytes in callus formation, the femurs of 20-week-old *Ng2-CreER;Rosa26R^tdTomato^* mice were fractured. One week before the fracture, the mice were intraperitoneally injected with tamoxifen three times. After three weeks, femurs were collected and histologically analyzed. Several clumps of tdTomato (Ng2)-expressing cells were observed in the newly formed callus (Figure 5A), and these cells expressed Runx2 and type 1 collagen (Figure 5B), suggesting that the endogenous pericytes also differentiated into osteogenic cells and contributed to bone fracture healing.

## 3. Discussion

In the present study, we successfully isolated pericytes from mouse embryos at 14.5–16.5 dpc. As pericytes are known to be distributed around small blood vessels, pericytes can be isolated from various organs. Although several previous studies regarding pericyte isolation have been reported, as shown in Table 1, precise markers for pericytes have not been determined. Most studies have isolated CD146-positive cells as pericytes. However, the fact that CD146 is also expressed in MSCs [14] raises the question of whether a genuine cell population of pericytes was isolated in these studies. NG2 is an important marker of pericytes. Although NG2 is not a completely specific pericyte marker, it is not expressed in MSCs [9,16]. To isolate a more homogenous population of pericytes, we isolated cells expressing both CD146 and NG2 while lacking CD31, CD45, and Ter119. Our isolated pericytes also had high expression of *Pdgfrb*, which is another important pericyte marker (Figure 1B). Our sorting methods may be one of the most recommendable methods for isolating pericytes while excluding MSCs. Although the pericytes were isolated from mouse embryos in this study, further investigation is needed to demonstrate whether the cell population can be obtained from tissues of adult mice.

There are still many discussions regarding the multilineage potential of pericytes. Many previous studies have demonstrated that pericytes possess the capacity to differentiate into multiple cells of mesenchymal lineages, especially myogenic, adipogenic, and fibrogenic lineages, both in vitro and in vivo [13,14,15]. On the other hand, Guimarães-Camboa et al. performed lineage-tracing experiments using the *Tbx18-CreERT2* mouse line and demonstrated that endogenous pericytes did not differentiate into other cell lineages in vivo [27]. In the present study, we showed that our isolated pericytes have a trilineage differentiation capacity in vitro (Figure 2), and a lineage-tracing study using the *Ng2-Cre* or tamoxifen-inducible *Ng2-CreER* mouse line demonstrated that endogenous pericytes can differentiate into osteogenic cells in vivo (Figure 3 and Figure 5). The discrepancy in these results is likely due to the lack of absolutely specific markers for pericytes. In addition, the relationship between pericytes and MSCs remains controversial. Some studies have speculated that MSCs are derived from pericytes [9]. Other studies have suggested that pericytes originate from MSCs [28,29]. Although we believe it is possible that pericytes are de facto different types of cells than MSCs, the identity, ontogeny, and progeny of both cell types should be addressed in further studies.

As pericytes are involved in various physiological functions, pericytes can contribute to the regeneration of various tissues in response to injury [30]. The formation of connective tissue is common to many injuries and pathologies. Several previous studies have reported that a specific subtype of pericytes turns into type 1 collagen-producing fibroblasts after acute injury [31,32] and participates in the formation of a fibrotic scar in the healing process of wounds or skeletal muscle injuries [33]. In spinal cord injury, pericytes also participate in the formation of scar tissue after injury. Göritz et al. demonstrated that pericytes were recruited into an injured spinal cord and gave rise to scar-forming stromal cells. The authors also showed that blocking the generation of pericyte progeny resulted in failure to seal the injured tissue [34]. As pericytes have myogenic differentiation potential, pericytes can influence skeletal muscle pathophysiology [35]. Several previous studies have demonstrated that in cases of acute or chronic muscle injury, pericytes generated myofibers to repair the damaged muscle [24,36]. Richardson et al. also showed that adipose tissue pericytes can convert into adipocytes in response to thermal injury [37]. Our current study also showed that in response to bone fracture, pericytes were recruited to the fracture site and differentiated into osteogenic cells to contribute to bone fracture healing.

## 4. Materials and Methods 

### 4.1. Pericyte Isolation and Culture

Mouse embryos were obtained from a pregnant wild-type mouse at the E14.5–16.5 stage. After the head, internal organs, and skin were removed, the embryo’s skeleton was incubated in 0.25% trypsin solution for 15 min at 37 °C. During the incubation, the solution was strongly shaken every 5 min. After being pipetted up and down, the solution was transferred to a new tube through a 40 μm cell strainer (Corning Inc., Corning, NY, USA). After adding culture medium (Dulbecco′s Modified Eagle Medium: DMEM, Fujifilm-Wako, Tokyo, Japan) to neutralize the trypsin, the cell solution was centrifuged at 500× *g* for 5 min. After the supernatant was aspirated, the cell pellet was resuspended in DMEM. The resuspended cells were seeded on 10 cm plates and incubated overnight at 37 °C in a humidified 5% CO_2_ environment. The next day, the adherent cells were detached with a 0.25% trypsin/EDTA solution. After adding the culture medium, the cell solution was centrifuged at 500× *g* for 5 min. After the supernatant was aspirated, the cell pellet was resuspended in ice-cold Hank’s Balanced Salt Solution (HBSS) containing 2% fetal bovine serum (FBS) and 1% penicillin/streptomycin. Then, the solution was stained for 30 min on ice with the following antibodies: phycoerythrin (PE)-conjugated anti-NG2 (Miltenyi Biotec Bergisch Gladbach, Germany, for pericytes), allophycocyanin (APC)-conjugated anti-CD146 (BioLegend, San Diego, CA, USA, for pericytes), PE-Cy7-conjugated anti-CD31 (BD Pharmingen, San Diego, CA, USA, for endothelial cells), PE-Cy7-conjugated anti-CD45 (BD Pharmingen, for hematopoietic cells), and PE-Cy7-conjugated anti-Ter119 (BD Pharmingen, for erythroid cells). Propidium iodide (PI, 2 μg/mL, Sigma-Aldrich, Tokyo, Japan) was used to eliminate dead cells. To isolate a cell population of pericytes, NG2+, CD146+, CD31−, CD45−, and Ter119− cells were sorted using a BD FACSAria II (Becton Dickinson, Franklin Lakes, NJ, USA) or Moflo (Beckman Coulter Inc. Brea, CA, USA) (Figure 1A). Analysis of the sorted cell populations routinely demonstrated 99% purity. After sorting, the sorted NG2+ and CD146+ cells were seeded on 48-well plates and cultured in DMEM supplemented with 10% FBS.

### 4.2. Immortalization of Pericytes

To establish an immortalized pericyte line, SV40 large T antigen was stably transduced into the isolated pericytes using a retroviral transduction system. The pMSCV-SV40 large T vector was kindly gifted by Takuro Nakamura (The Cancer Institute, Japanese Foundation for Cancer Research (JFCR), Tokyo, Japan). In addition, to label the implanted pericytes, GFP (pMXs-GFP vector) was stably transduced into the immortalized pericytes using the same retroviral transduction system.

### 4.3. Osteogenic Differentiation Assay

Pericytes were seeded in 24-well plates at 1 × 10^4^ cells/well. Cells were cultured in Minimum Essential Medium Eagle (α-MEM, Wako) containing 10% FBS, 50 μM ascorbic acid 2-phosphate (Sigma-Aldrich), 5 mM disodium β-glycerophosphate (Sigma-Aldrich), 100 nM dexamethasone (Sigma-Aldrich) and 0.3 mg/mL recombinant human BMP-2 (R&D Systems, Minneapolis, MN, USA) for 6–9 days. The culture medium was changed every 3 days, and ALP activity and mineralization potential were evaluated by an ALP assay and von Kossa staining, respectively, as previously described [38].

### 4.4. Adipogenic Differentiation Assay

Pericytes were seeded in 24-well plates at 4 × 10^4^ cells/well and cultured with Mesenchymal Stem Cell Basal Medium (MSCBM, Lonza, Basel, Switzerland, #PT-3238) until the cells became 100% confluent. After reaching confluence, the medium was changed to adipogenic induction medium (Lonza, #PT-3102B) supplemented with h-insulin, L-glutamine, mesenchymal cell growth supplement (MCGS), dexamethasone, indomethacin, isobutylmethylxanthine (IBMX), and GA-1000 (Lonza, #PT-4135) for 4 days. Then, the cells were cultured in adipogenic maintenance medium (Lonza, #PT-3102A) supplemented with h-insulin, L-glutamine, MCGS, and GA-1000 (Lonza, #PT-4122) for 3 days. The above processes of induction for 4 days and maintenance for 3 days were repeated 3 times to stimulate adipogenic differentiation. Noninduced control cells were cultured with only the supplemented adipogenic maintenance medium on the same schedule. To evaluate the adipogenesis of pericytes, quantitative PCR analysis and Oil Red O staining were performed.

### 4.5. Chondrogenic Differentiation Assay

Pericytes were aliquoted into 15 mL polypropylene culture tubes at 1 × 10^6^ cells/tube. The cells were centrifuged at 150× *g* for 5 min at room temperature. The centrifuged cells were incubated with chondrogenic induction medium (Lonza, #PT-3925) supplemented with dexamethasone, ascorbate, ITS (insulin, transferrin and selenium), GA-1000, sodium pyruvate, proline, L-glutamine (Lonza, #PT-4121), 5 ng/µL TGF-β3 (Lonza, #PT-4124), and 100 ng/µL BMP-6 at 37 °C for 4 weeks. The medium was changed every 2–3 days. After 4 weeks of culture, half of the pellets were fixed with 4% paraformaldehyde, embedded in paraffin, and cut into 5 µm sections for alcian blue staining. The other half of the pellets was used for quantitative PCR analyses.

### 4.6. Experimental Animals

*Ng2/Cspg4-Cre* [39], *Ng2/Cspg4-CreER* [40], and *Rosa26R^tdTomato^* [41] transgenic mice were purchased from Jackson Laboratory (Bar Harbor, ME, USA, Stock Nos: 008533, 008538, and 007909, respectively). *BALB/cAJcl-nu/nu* mice were purchased from CLEA Japan Inc. (Tokyo, Japan). *Ng2-Cre;Rosa26R^tdTomato^* mice and *Ng2-CrER;Rosa26R^tdTomato^* mice were generated by crossing these mouse lines for in vivo lineage-tracing studies. Eight-week-old *Ng2-CreER;Rosa26R^tdTomato^* mice were administered tamoxifen (Sigma-Aldrich, 1 mg/10 g mouse body weight) intraperitoneally every other day for a total of three times to activate the transgene.

### 4.7. Bone Fracture Model and Pericyte Implantation

Femurs of *BALB/cAJcl-nu/nu* mice or tamoxifen-injected *Ng2-CreER;Rosa26R^tdTomato^* mice were used for the fracture model. We first opened a knee joint of the mice using a razor blade, cut the collateral ligaments, pushed the patella aside, and bent the joint 90°. Then, a 27 G needle was inserted into the intramedullary space from the distal side of the femur. After the insertion, the femur was fractured at the center of the bone. When the femurs of *BALB/cAJcl-nu/nu* mice were fractured, a 1 × 10^6^ cells/100 µL Matrigel solution (Corning Inc.) of the GFP-expressing immortalized pericytes was implanted in the defect site. X-ray images were taken every week after the fracture to observe callus development. Three weeks after the fracture, the femurs with a callus were harvested for further analyses. These animal experiments were conducted with approval from the Animal Study Committee of Tokyo Medical and Dental University (latest identification code: A2018-163C4, approved on October 10, 2018) and conformed to the relevant guidelines and legislations.

### 4.8. Quantitative Real-Time PCR (qPCR)

Total RNA from the cultured or differentiated cells was extracted using the miRNeasy Mini Kit (Qiagen, Hilden, Germany) or TRIzol (Thermo Fisher Scientific Inc., Waltham, MA, USA) according to the manufacturer’s instructions or as previously described [42]. The RNA quality and quantity were measured using a NanoDrop 2000 spectrophotometer (Thermo Fisher Scientific Inc.). To analyze the expression of pericyte markers or chondrogenic and adipogenic markers, RNAs were reverse-transcribed using ReverTra Ace, and qPCR was performed using the Brilliant III Ultra-Fast SYBR Green QPCR Master Mix (Agilent Technologies, Santa Clara, CA, USA). The following primers were used: *Gapdh*, *Ng2*, *Mcam/CD146*, *Pdgfrb*, *Adipoq*, *Pparg*, *Col2a1*, *Sox9*, and *Acan*. *Gapdh* was used as an internal control. All qPCRs were performed using the Mx3000P real-time PCR system (Agilent Technologies), and the ΔΔCt method was used for data analysis.

### 4.9. Histological and Immunohistochemical Analysis

Femurs from the above fractured mice or *Ng2-CreER;Rosa26R^tdTomato^* mice were fixed in a 4% paraformaldehyde solution for 1 h and then incubated in a 25% sucrose solution overnight. Then, the samples were embedded in 4% carboxymethyl cellulose (CMC, Leica Microsystems, Tokyo, Japan) and frozen in liquid nitrogen. The embedded samples were cut into 7 µm sections by a cryostat (CryoStar NX70, Thermo Fisher Scientific Inc.). The distribution of GFP or tdTomato-expressing cells in the newly developed callus was investigated with a fluorescence microscope (BZ-9000; Keyence, Osaka, Japan). Immunohistochemistry analyses were also performed on frozen sections as previously described [43]. Antibodies against NG2 (Miltenyi Biotec, #130-097-455), PDGFRβ (Abcam, Cambridge, UK, #ab32570), CD31 (BD Pharmingen, #550274), ALP (kindly gifted by Dr. Kimimitsu Oda, Niigata University Graduate School of Medical and Dental Sciences, Niigata, Japan), Osterix (Santa Cruz Biotechnology, Inc., Dallas, TX, USA, #sc-22536-R), Sost (R&D systems, #AF1589), Runx2 (R&D systems, #MAB2006), and type 1 collagen (Abcam, #ab34710) were used as primary antibodies. Stained sections were observed using a fluorescence microscope or a confocal microscope (A1R; Nikon, Tokyo, Japan).

### 4.10. Statistical Analyses

All the data are presented as means ± SDs. Differences between groups were analyzed by Student’s *t*-test. The values were considered significant at *p* < 0.05. The results are representative of more than three individual experiments.

## 5. Conclusions

In conclusion, we established a novel immortalized pericyte line and showed its osteogenic differentiation potential in vitro and in vivo. Furthermore, in vivo pericyte-lineage-tracing studies have demonstrated that endogenous pericytes also differentiate into osteoblasts and osteocytes and contribute to bone fracture healing as a source of osteogenic cells. Bone fracture is one of the most frequent problems in the orthopedic field around the world. As pericytes are abundant in animals and play diverse critical roles in tissue repair, pericytes can be a promising therapeutic candidate for tissue regeneration. Our study demonstrated a significant contribution of injected pericytes to bone fracture healing. Local injection of pericytes may be useful for treating bone fractures with a delayed union or nonunion as well as bone diseases causing bone defects. To achieve the clinical application of pericytes, further investigations will be needed.

## Figures and Tables

**Table 1:** Methods for pericyte isolation.

Isolation Method	Markers for Sorting	Species	Tissue	Year	Ref.
FACS *	CD146+, CD34−, CD45−, CD56−	Human	Placenta	2011	[17]
FACS	CD146+, CD34−, CD45−, CD56−	Human	Hepatic tissue	2012	[18]
Magnetic beads	CD146+	Human	Umbilical cord	2013	[19]
FACS	CD146+, CD34−, CD45−, CD56−	Human	Skeletal muscle	2014	[20]
FACS	CD146+, CD31−, CD34−, CD45−	Human	Adipose	2016	[21]
FACS	CD146+, CD34−, CD45−, CD56−, CD144−	Human	Cardiac tissue	2016	[22]
FACS	CD146+, CD34−, CD45−	Human	Canine adipose	2017	[23]
FACS	NG2/DsRed+, Nestin/GFP+/−	Mouse	Skeletal muscle	2017	[24]
FACS	CD146+, CD34−, CD45−, CD56−	Mouse	Hepatic tissue	2018	[25]
FACS	CD13+, CD31−, CD41−, CD45−	Mouse	Brain	2018	[26]

* FACS: Fluorescence-activated cell sorting.
